# Mule Bite to the Male Genitalia with Complete Penile and Anterior Urethra Amputation: Unusual Case and Review of the Literature

**DOI:** 10.5402/2011/723154

**Published:** 2011-04-19

**Authors:** M. A. Lakmichi, B. Wakrim, R. Jarir, Z. Dahami, M. S. Moudouni, I. Sarf

**Affiliations:** Urology Division, Mohammed the VIth University Hospital, Faculty of Medicine, Cadi Ayyad University, Marrakesh, Morocco

## Abstract

Animal bite is rare with few cases reported in the literature. The morbidity of animal bites is directly related to the severity of the initial wound. Most victims are boys, and dog bites are the most common injury. Infectious complications are unusual, since treatment is sought early (Wein 2007). Thus, urologists are not usually familiar with management and principles for treating this condition. The authors report the case of a 38-year-old male with a severe mule bite injury to the genitalia causing complete penile and anterior urethra amputation and scrotal wound with no involvement of its contents. To our knowledge, no such case had ever been reported in the medical literature. This kind of emergencies is challenging for urologists.

## 1. Introduction

Animal bite is rare with few cases reported in the literature. The morbidity of animal bites is directly related to the severity of the initial wound. Most victims are boys, and dog bites are the most common injury. Infectious complications are unusual, since treatment is sought early [1]. Thus, urologists are not usually familiar with management and principles for treating this condition. The authors report the case of a 38-year-old male with a severe mule bite injury to the genitalia causing complete penile and anterior urethra amputation and scrotal wound with no involvement of its contents. To our knowledge, no such case has ever been reported in the medical literature.

## 2. Case History

**Figure 1:**
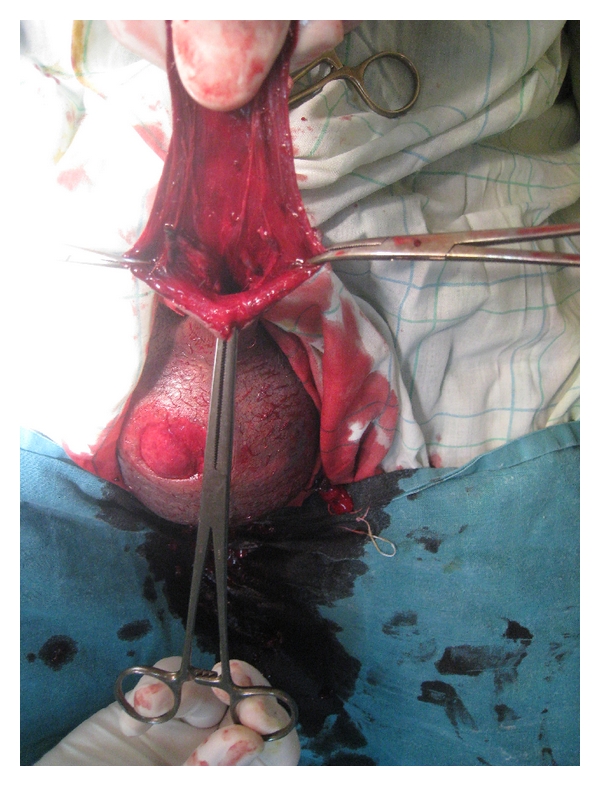
Aspect of the penoscrotal trauma at presentation (note the remaining penile shaft and the right hemiscrotum injury).

**Figure 2:**
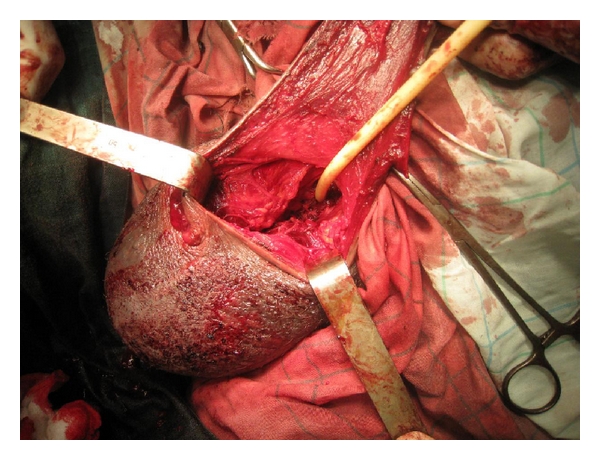
Extent of penile amputation after haemostasis (note the Foley catheter inserted at the end of urethra).

A 38-year-old countryman, married and father of a young girl, with no past medical history was referred to our department for evaluation after an attack by his mule. At presentation, a complete amputation of the penis with a wide haematocele was noted (Figures 1 and 2). The wound had sharp edges. The patient was in good hemodynamic condition despite the important bleeding reported by his family before admission. The testis was palpable in the scrotum. 

The scrotal lesion was classified as Grade II according to the American Association for the Surgery of Trauma organ injury scale, when penile trauma was classified as Grade V following the same injury scale [2].

**Figure 3:**
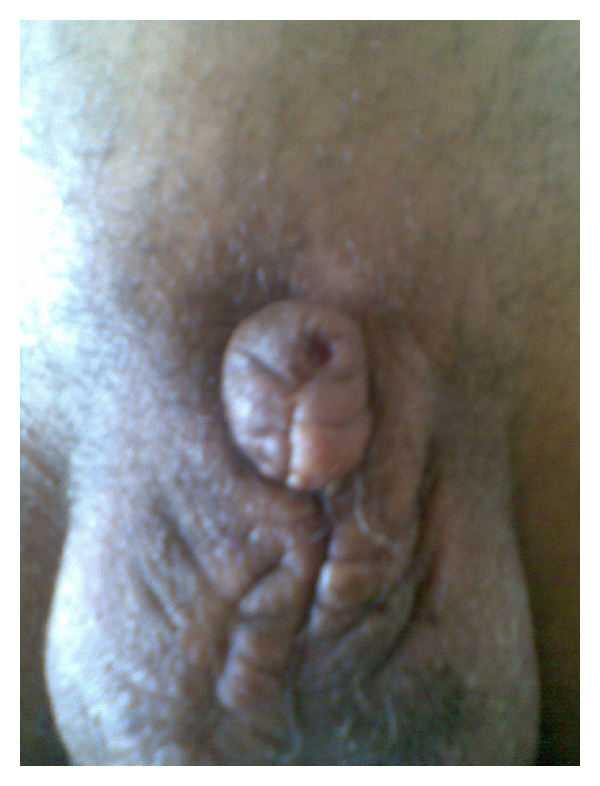
Phalloplasty with final aspect after 12 weeks.

Intravenous ampicillin sodium and sulbactam sodium were administrated. Passive tetanus and rabies immunization was undertaken. After brief debridement and copious irrigation of the wound with saline and povidone-iodine solution, the patient had an ultrasound that showed the testes and epididyms of normal appearance. In the operating room and under a general anaesthesia, the wound was irrigated with thorough debridement. Scrotal wound at the right hemiscrotum was noted (Figure 1). A huge haematocele infiltrated the entire perineal area. Surgical exploration was undertaken to ensure testicular integrity. Both hemiscrotums were explored. A wide right haematocele was present, and careful examination of the testis and epididymes and spermatic cord showed no anomalies. Contralateral exploration was performed to evaluate conditions in the left testis. No signs of trauma were noted. Thereafter, we found a complete pullout of all erectile bodies: the corpora spongiosum, cavernosa at their bony insertions, and whole anterior urethra. Profuse bleeding occurred after removal of the huge hematoma at the insertion of the corpora cavernosa for a full exploration. Therefore, we decided to proceed to catheterization of the urethra at its membranous part (the remaining distal end of the urethra) by a Foley catheter. The haemostasis at the insertion of the penis was then performed by stitches. We proceeded to the placement of a Penrose drain. The scrotal wound was closed, since there was no sign of infection. A cystotomy catheter was inserted with a compression bandage to the perineum. A phalloplasty was performed from the remaining penile shaft with a scrotal flap (Figure 3). However, in a second step, a final phalloplasty will be performed with a groin flap. A penile prosthesis will then be implanted. The Penrose drain was removed on the second postoperative day, and the patient was discharged on the seventh postoperative day, when no signs of infection were noted. Wide-spectrum antibiotics and analgesics were maintained for 7 days. Psychological support was provided to the patient by psychiatric consultations first in our urology division, then, after being discharged. 

A perineal urethrostomy was planned, with the consent of the patient, after complete resorption of the hematoma.

## 3. Discussion

Genital trauma due to animal bite is rare. Wolf et al. presented 4 new cases of dog bite and reviewed 4 previously reported cases [3]. Cummings and Boullier reported on 8 patients treated for dog bite of the scrotum [4]. However, in the largest series in the literature, Gomes et al. reported 10 new cases. Of the 2 men and 8 boys 8 were attacked by dogs, 1 by a horse, and 1 by a donkey, respectively [5]. But no patient of this series has been severely bitten by a mule. Dog bites are a common form of trauma in the United States with an incidence of 12.9/10,000 individuals. Children have a 3.2-fold higher bite rate than adults [4]. 

Various injury types have been reported in the literature. Kyriakidis et al. described partial amputation of the penis due to a dog bite [6], while Piza-Katzer and Latal reported a case of penile skin loss [7]. Donovan and Kaplan treated amputation of the cord and amputation of the glans in 1 case each [8]. The injuries reported by Wolf et al. included testicular loss in 2 of their 4 cases [3]. Gomes et al. had 5 patients who presented with minimal or no skin loss, including 2 with urethral lacerations. There was moderate-to-extensive tissue loss in 5 patients, including degloving penile injury in 2, traumatic spermatic cord amputation in 1, complete penile and scrotal avulsion in a 5-month-old infant, and partial penectomy in 1 [5]. Nevertheless, no complete penile and anterior urethra amputation has been reported in adults. 

Management of such injuries comprises debridement of devitalized tissue and copious wound irrigation with saline and antiseptic solutions. When there is no infection, immediate primary closure along with prophylactic broad-spectrum antibiotics is performed [1]. 

In our patients, the remaining penile shaft closed the wound decreasing the important bleeding flow immediately after the animal bite. Thereafter, blood infiltrated scrotum and perineum explaining the huge hematoma noted at presentation. Usually, haemostasis is easily performed for such injuries. But in our patient, this part of the surgical management was the most difficult. The deepness of the operating field and the important bleeding flow (Figures 1 and 2) made stitches very difficult to make. Thereafter, the Foley catheter (Figure 2) facilitates the insertion of stitches around the remaining end of the urethra, not far from prostatic apex. Thus, the Foley catheter once inserted helped prevent haemostasis stitches to close urethra lumen. 

The therapy depends on the type of injury. Smaller wounds can initially be treated through wound dressing or suturing, after cleaning. Extensive injuries should initially be treated conservatively. If the wound remains uninfected, reconstruction should follow later, but after removing possible necrotic tissue. Particularly in cases of partial penile amputation, or in the rare event of unilateral or bilateral testicular loss, primary reconstruction should be attempted after removing any existing necrosis [9].

Moreover, in any penile amputation injury, possible urethral involvement must be considered, and this requires an adequate diagnosis. The long-term followup of urethral injuries from animal bites shows the worst results, with urethrocutaneous and urethroscrotal fistula or recurrent urethral strictures. Therefore, some authors postulate that injuries to the urethra should be treated later, when it can safely be assumed that there is no inflammation in the sensitive urethral tissue [5].

One of the main problems in bite wounds is the risk of infection, which often occurs in the first 48 h after the injury. This risk is up to 30% in uncomplicated wounds [9]. After removing dead tissue and antiseptically cleaning the wound, a broad-spectrum antibiotic is obligatory, even if there is no firm evidence of pathogenic agents. In addition, the vaccination status should be verified; if such protection is insufficient, then vaccination against tetanus (or rabies) should be immediate. The most frequent bacterial species are *Staphylococcus*, *Streptococcus*, *Escherichia coli*, and anaerobes [10].

## 4. Conclusion

Although rare, genital trauma caused by animal bite is a potentially severe condition with distinctive characteristics. Irrigation and debridement of dead tissue is the cornerstone of treatment, and they should always be performed. Primary closure is possible in most cases and usually achieves good functional and cosmetic results. Wide-spectrum antibiotics prophylaxis is recommended in all cases. Systemic diseases potentially transmitted by animals must be considered, and prophylaxis should be administered accordingly. Urologists should be knowledgeable of these kinds of emergencies. Thereafter, the extraordinary emotional situation of the patient must be considered, and a psychiatric supports should be provided.
